# Medical Reminiscences of Forty Years Ago

**Published:** 1898-11

**Authors:** G. G. Roy

**Affiliations:** Atlanta, Ga.


### MEDICAL REMINISCENCES OF FORTY YEARS AGO.

By G. G. ROY, M.D.,

ATLANTA, GA.

When typhoid fever first developed at Tide Water, Virginia, I have heard my father, Dr. A. G. D. Roy, say that it was a hard matter to diagnose it from the adynamic forms of severe malarial fever not uncommon in that section, and the two diseases requiring entirely different modes of treatment, many patients were lost that might have been saved had the distinction been clearly defined and understood by the physicians then and the treatment changed. The physicians at that time had never met with a case of typhoid fever, but had been accustomed to treat *typhus* and malarial fevers in all their insidious and multiform types, which required (as has been called) *heroic treatment.* When this new disease appeared, they thought it another form of fever, but of malarial origin, and began the same heroic treatment; the result was their patients survived only a few days under that treatment. I very well recollect how my father told me he began the treatment successfully by a species of practical *induction.*

In a certain family, one of the best neighboring physicians, a very successful practitioner in malarial fevers with the heroic plan, had eight or ten cases of fever among the negroes on the plantation, and in a week or ten days every one died.

My father was then called to attend the other negroes and members of the family with the same fever; and learning the mode of treatment of Dr. G., who attended those that died, determined to try the opposite plan of treatment—*i. e.* little medicine, nourishment and stimulants, and leave to nature the cure. Under this treatment all of his patients recovered. Then, for the first time, it dawned upon the physicians in that section that they had a new fever to deal with, one which could not be aborted or its career shortened with quinine and calomel, and that it would "run its course," terminating on the seventh day, or on some multiple of that number, and that a supporting treatment was most likely to bring success. Typhoid fever of that day, it appears to me, was more severe, but more successfully combated or managed than now. I don't know the reason, unless it is that mankind has physically degenerated, and is thereby the less able to resist and throw off diseases.

It appears to me that recovery took place oftener after serious brain complications and intestinal hemorrhage then than now.

The mode of treatment was somewhat similar then and now; small doses of calomel were given to stimulate the secretions of the liver, at intervals during the progress of the disease; one-eighth to one-sixth grains tonic and stimulating doses of quinine. Turpentine emulsion and (what will strike the younger members of the profession with holy horror) fly-blisters to the scalp in brain complications and over the abdomen as soon as extreme tenderness developed.

I am a living monument to this plan of treatment. When about thirteen or fourteen years of age, after a week or two of malaria, during which I kept up and attended school, though unfit for study, I took my bed on the 15th day of July. About the 20th I became unconscious and have no recollection of any thing that transpired until late in the following September, when I regained consciousness.

During this time my scalp was shaved and a blister applied, and as soon as this "dried up" another was applied, and the scalp was kept a running sore for six or eight weeks. At the same time I was blistered, cupped and leeched over the abdomen, and these were persisted in like the blister to the scalp. I do not mean to say that I was cupped and leeched on the blistered surface, but the cupping and leeching were used alternately with the blisters, and I bear the marks of the scarificator on my body to this to-day.

Now, the doctors of this age will say, that I must have been a horse or a mule to stand such cruel treatment, but without it, I honestly believe that I would not be living to-day. My father's rule was to have me given two to four tablespoonfuls of milk and mush every two hours, day and night. This was prescribed as medicine, and given with the same regularity when I could be made to swallow. I have no doubt but that I was supported by rectal administrations also.

My case was given up as hopeless by my father and all the other physicians in attendance, but my old colored nurse, all these long and dreary days and nights, lay by my bedside; and it was to her that the first dawn of returning consciousness was revealed. She told me that one night at 12 o'clock, when my father had retired, heart-sick, worn-out and hopeless, that I spoke. She gave heed to the sound which she could hardly believe, and was greatly startled. I recognized her and said: "Harriet! I am as hungry as a wolf. Have you anything to eat?" She said: "No, Marse Gus! You haven't eaten anything for a long time; and there is nothing to eat in the room." I then said: "Have you any ice?" She gave me a piece, and said I cracked it with my teeth "like a pig cracking corn."

Of course, the history of my sickness was derived from my father and my colored nurse, who had nursed me when a baby.

There was a mute (deaf and dumb) gentleman in the neighborhood who was very fond of the family, and especially so of me. He was a vigilant and competent nurse also. I was told he would come to stay with me nearly every night. After I recovered he thought he had a good joke on me. By his language of signs, he thought it very funny to tell my acquaintances that one night in holding the vessel for me to urinate, the urine fell upon his hand and burnt him so badly that he dropped the vessel. This he thought was a good joke on me, and he would laugh himself inordinately when telling it.

When I began to convalesce, I was indeed a pitiable object to behold; the bones of my ankles, knees, hips and elbows were "through the skin." I had forgotten how to walk, and had to learn it over again like a baby, and my bald head made me an object so frightful that I was scared of myself the first time I looked into a mirror, and did not repeat it for many weeks afterwards.

I shall continue this subject in the next issue of THE JOURNAL, giving some of my early experiences in treating this formidable disease.

